# Design, Screening, and Impact of sgRNAs Targeting Bovine Prolactin Gene Receptor on Embryonic Development Using Stably Transfected Cell Lines

**DOI:** 10.3390/biology14040425

**Published:** 2025-04-15

**Authors:** Daqing Wang, Guifang Cao, Xin Li, Xin Cheng, Zhihui Guo, Lu Li, Hong Su, Kai Zhang, Yuanyuan Zhang, Min Zhang, Feifei Zhao, Yifan Zhao, Junxi Liang, Yiyi Liu, Yong Zhang

**Affiliations:** 1 College of Veterinary Medicine, Inner Mongolia Agricultural University, Hohhot 010011, China; wangdaqing050789@126.com (D.W.); guifangcao@126.com (G.C.); 17647628761@163.com (X.L.); 17822106812@163.com (X.C.); h1454738358@163.com (Z.G.); yunsong-5410@163.com (L.L.); hongsu1995@126.com (H.S.); zhangkai040423@163.com (K.Z.); zyyworkaccount@163.com (Y.Z.); zhangmin5400@126.com (M.Z.); imauzff@126.com (F.Z.); yifanz311@163.com (Y.Z.); ljx2223128@163.com (J.L.); 2 Animal Embryo and Developmental Engineering Key Laboratory of Higher Education, Institutions of Inner Mongolia Autonomous Region, Hohhot 010011, China; 3 Inner Mongolia Autonomous Region Key Laboratory of Basic Veterinary Medicine, Hohhot 010011, China; 4 College of Life Sciences, Inner Mongolia University, Hohhot 010021, China; 5 College of Life Sciences, Inner Mongolia Agricultural University, Hohhot 010011, China

**Keywords:** PRLR, gene editing, somatic cell nuclear transfer

## Abstract

This study designed three sgRNAs (sgRNA139, sgRNA128, and sgRNA109) targeting the prolactin gene receptor (PRLR) in fetal cattle, utilized Cas9 to cleave endogenous DNA, and screened stable cell lines for somatic cell nuclear transfer experiments to investigate the impact of different editing sites on embryonic development. The results showed that sgRNA139 had the highest cleavage efficiency (Fcut = 0.65, Indels = 42.19%), while sgRNA109 had the lowest (Fcut = 0.45, Indels = 35.31%). No significant differences were observed in cell growth status after electroporation (*p* > 0.05), and the transfection efficiency exceeded 90% after five days of culture. In the evaluation of key embryonic development indicators, sgRNA109 significantly reduced the cleavage rate and blastocyst rate (*p* < 0.01), whereas sgRNA139 showed no significant effect on the cleavage rate (*p* > 0.05), but its blastocyst rate was slightly lower than that of the control group (*p* > 0.05). This study demonstrates that highly specific sgRNAs and stable edited cell lines used as donor cells can significantly regulate the later stages of embryonic development. This study not only provides new experimental evidence for the functional study of the PRLR but also lays an important theoretical foundation for the innovation of molecular breeding technologies in dairy cattle.

## Simple Summary

In this study, three specific sgRNAs (sgRNA139, sgRNA128, and sgRNA109) targeting exon 9 of the prolactin gene receptor (PRLR) in fetal cattle were designed. DNA cleavage was mediated using the CRISPR/Cas9 system, and stable cell lines were screened. Through somatic cell nuclear transfer technology, the effects of different editing sites on embryonic development were systematically investigated. The results showed that sgRNA139 exhibited the highest DNA cleavage and repair efficiency. In terms of embryonic development indicators, sgRNA109 significantly reduced the cleavage rate and blastocyst rate (*p* < 0.01), while sgRNA139 had no significant effect on the cleavage rate (*p* > 0.05). These findings confirm that highly specific sgRNAs and their stable edited cell lines, as donor cells, can significantly regulate the later stages of embryonic development. This study provides important theoretical foundations and practical guidance for gene editing and molecular breeding in dairy cattle.

## 1. Introduction

In recent years, the consistent increase in global temperatures has not only impacted human populations but also posed a threat to the continued growth of livestock production. Within the dairy industry, potential heat stress—calculated using temperature and humidity data exceeding specific comfort thresholds—has been escalating worldwide. This issue affects dairy cow productivity and reproductive performance and, in extreme cases, can lead to livestock mortality [1]. Consequently, animal husbandry must proactively implement various strategies to mitigate both current and future economic losses. Previous studies have identified several genes associated with variations in heat tolerance among dairy cattle, including prolactin gene receptor (PRLR), super oxide dismutase1 (SOD1), heat shock protein family B (small) member 7 (HSPB7), heat shock factor 1 (HSF1), heat shock protein 90 alpha family class B member 1 (HSP90AB1), heat shock protein family B (small) member 6 (HSPB6), myosin IA (MYO1A), and Tectonin beta-propeller repeat containing 2 (TECPR2) [2,3,4,5].

Prolactin gene receptor (PRLR) is a protein situated on the cell surface, and has a molecular structure comprising an extracellular domain, a transmembrane domain, and an intracellular domain [6]. When prolactin (PRL) binds to PRLR, it induces a conformational change in the receptor and subsequently activates intracellular signaling pathways. This activation leads to modifications in various physiological processes, including cell proliferation, differentiation, metabolism, and secretion, thus regulating the physiological changes induced by heat stress [7].

To date, six SLICK phenotypes from natural mutations causing truncated PRLR have been identified, though the specific mutation locations vary. At present, most in-depth research has focused on SLICK1 and SLICK2. However, recent studies have extended the scope of research to SLICK6. SLICK1 was discovered in Senepol cattle [8]: the SLICK1 mutation, which is a variation in the prolactin receptor (PRLR) gene, occurs within the PRLR region on bovine chromosome 20 (BTA20) [9]. This mutation leads to the deletion of certain exons of the PRLR, thereby disrupting the prolactin signaling pathway. SLICK2 was found in both Carora and Limonero breeds. Limonero cattle also exhibited SLICK3 [10], which can be widely utilized to enhance heat tolerance in dairy cattle in tropical regions. Later, three new SLICK phenotypes from new mutation sites in PRLR were identified and designated SLICK4–SLICK6. SLICK4 was identified in Criollo Lechero Tropical cattle, while SLICK5 mutations were present in Blanco Orejinegro, Costeño con Cuernos, Hartón del Valle, Romosinuano, and Carora breeds. These four breeds also exhibited SLICK6 mutations [11]. SLICK1 through SLICK6 share common phenotypic traits. Animals carrying these mutations display short and sparse coats. This unique hair morphology enhances heat dissipation, improves thermoregulatory capacity, and enables animals to maintain more stable milk production during heat stress. China has lagged behind in terms of research into the heat tolerance mechanisms of these mutations. In addition, there is a significant deficiency in screening for gene editing sites related to the heat tolerance of dairy cattle. This has limited progress in the autonomous breeding of gene-edited dairy cattle that are resistant to heat stress.

CRISPR-associated protein 9, abbreviated as Cas9, hails from the bacterial CRISPR–Cas system. This system is an adaptive immune defense mechanism that bacteria have developed over a long period of evolution, which is used to defend against the invasion of foreign nucleic acids such as bacteriophages. Scientists discovered that a specific sequence within the guide RNA (gRNA) can undergo base–complementary pairing with the target DNA sequence. Guided by the gRNA, Cas9 forms a complex that binds precisely to the target site. This enables Cas9 to cleave specific DNA sequences [12]. The discovery has unlocked extensive applications of Cas9 in the field of gene editing.

This study designed various sgRNAs targeting the 50–204 bp region of the ninth exon in bovine PRLR, guiding the Cas9 protein to induce cleavage of endogenous DNA for self-repair. It then screened for stably transfected cell lines containing these sgRNAs and investigated the effects of these cell lines on embryonic development through SCNT experiments. The objectives were to establish new targeted sites for gene editing related to heat tolerance, develop new animal models for heat stress resistance, accelerate trait improvement, and lay the foundation for the breeding of heat-stress-resistant dairy cattle through gene editing.

## 2. Materials and Methods

### 2.1. Oocytes for Experiments

A total of 122 adult high-quality female cows were selected from the North Asia Youth Holstein Dairy Farm in Hohhot, Inner Mongolia Autonomous Region. After slaughter, fresh adult cow ovaries were collected and selected based on the following criteria: light red to dark red, shiny oval, smooth surface, length of about 2–4 cm, width of about 1.5–3 cm, thickness of about 1–2 cm, and high-quality bovine oocytes. A 20 mL syringe was used to suction and extract the zona pellucida, which should be complete, uniform, and smooth. The cytoplasm should be uniform and delicate and the granulosa cells should spread evenly and have a diameter of about 100–120 μm. Suitable cells were extracted and used for the in vitro maturation culture.

### 2.2. Materials and Instruments

IVM and IVC media were purchased from Stroebech Medi (Copenhagen, Denmark); ionomycin, 6-(Dimethylamino) purine, and hyaluronidase were purchased from Sigma-Aldrich (St. Louis, MO, USA); DPBS, fetal bovine serum, pancreatic enzymes, DMEM/F12, penicillin–streptomycin, and OPTI-MEM were purchased from Gibco (New York, NY, USA); DH5α competent cells were purchased from TAKARA (Shiga, Japan); T7 Endonuclease I was purchased from TransGen Biotech (Beijing, China); Bbs I Restriction Endonuclease was purchased from NEB (Ipswich, MA, USA); a Plasmid Extraction Kit was purchased from Omega Bio-Tek (Norcross, GA, USA); and pSpCas9(BB)-2A-EGF(PX458, plasmid#48138) was purchased from the Addgene website and stored.

We also used an Electrophoresis Apparatus (Bio-Rad, Hercules, CA, USA), gel imaging system (Gene, Houston, TX, USA), CO_2_ incubator cells, Centrifuge (Thermo, Waltham, MA, USA), and flow cytometer (SONY MA900, Tokyo, Japan).

### 2.3. Design of Experiments

**Figure 1:**
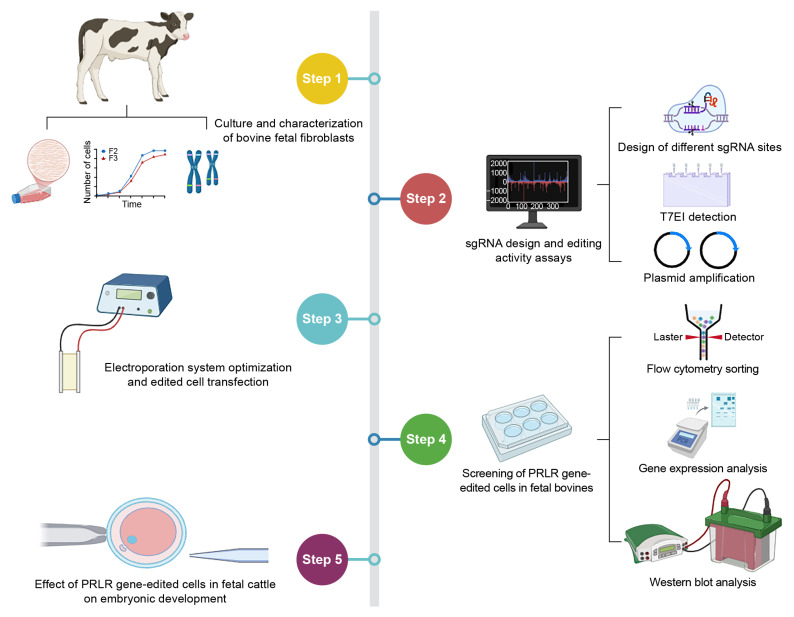
Experimental design.

The study design consisted of five parts. Step 1: fetal bovine fibroblast culture and characterization analysis; Step 2: sgRNA design and editing activity detection; Step 3: optimizing the electroporation system and editing the cells for transfection; Step 4: fetal bovine PRLR editing cell screening; and Step 5: assessing the effect of PRLR gene-edited cells in fetal cattle on embryonic development (Figure 1).

### 2.4. Fetal Bovine Fibroblast Culture and Characterization Analysis

#### 2.4.1. Fetal Bovine Primary Cell Lineage

The ear tissue block of the dairy cow was removed from the normal saline, 75% alcohol was added to soak the tissue block for 3–5 s each time, and the residual hair and impurities on the surface were quickly washed away. After washing, the ear tissue pieces were transferred to a clean Petri dish containing DPBS solution, the tissue pieces were cut to 1–2 mm^3^ with scissors and forceps, the minced cells were evenly spread at the bottom of the cell bottle, 3 mL of cell culture solution was added to moisten the cells, and the cells were placed in a 37 °C, 5% CO_2_ incubator. After 5–8 h of dry incubation, the cell flasks were cultured upright. The cells were incubated continuously for 10 days, and the cell growth morphology was observed every 24 h.

#### 2.4.2. Fetal Cattle Subculture

After 10 days of primary culture, we took out the cells, poured out the supernatant culture medium, added DPBS for 30 s–1 min, discarded the liquid after rinsing, added 2 mL of trypsin, and put them in an incubator at 37 °C with 5% CO_2_ to digest the cells for 1 min. We then suspended the adherent cells, added 4 mL of cell culture medium, terminated the digestion, put the liquid in a centrifuge at 1300 rpm for 3 min, and discarded it. Next, we added 1 mL of culture medium, resuspended the cells with a gun, and transferred them into a new Petri dish. The cells were subcultured for two passages in a row, and the third generation of cells (F3) was used for transfection experiments.

#### 2.4.3. Determination of Fetal Cattle Growth Curve

Cells passaged to passage 2 (F2) and passage 3 (F3) were seeded into 24-well plates, counted with hemocytometers 1, 2, 3, 4, 5, 6, 7, and 8 days after plate transfer, and the cell growth curves plotted.

#### 2.4.4. Analysis of Chromosomal Karyotype of Fetal Bovine Cells

F3 cells were transferred to 6-well cell culture plates, and continued to be cultured in a 37 °C, 5% CO_2_ incubator at 37 °C for 6~8 h. Then, the cells were isolated by trypsinization, followed by treatment with 0.075 mol·L-1 KCl hypotonic solution at 37 °C for 25 min, then fixed with a fixative solution for 8 min, centrifuged at 800 r·min^−1^ for 10 min; this process was repeated three times. The pre-chilled slides were tilted at 45 degrees dropwise to add the cell suspension, after which the slides were passed over an alcohol lamp, allowed to dry at room temperature, stained with Giemsa solution, and mounted with a mounting medium. Finally, we observed the number and morphology of chromosomes under a 100× oil microscope.

### 2.5. sgRNA Design and Editing Activity Assays

#### 2.5.1. sgRNA Design

In this experiment, referring to the PRLR mutation site, the 50–240 bp site of exon 9 of the PRLR in dairy cows was selected as the target region, and the target region was screened according to the principle of 18~20N+NGG by using the CRISPOR online website (http://www.rgenome.net/be-designer/ (accessed on 1 March 2024)). Three suitable guide RNAs (single guide RNAs, sgRNAs) were selected and named sgRNA109, sgRNA128, and sgRNA139, respectively (Table 1).

#### 2.5.2. CRISPR/Cas9 Editing to Construct Eukaryotic Expression Vectors

In this experiment, the pSpCas9(BB)-2A-EGF (PX458, plasmid#48138) vector was selected as the targeting vector. The oligonucleotide sequence corresponding to the target sequence (Table 2) was designed and sent to Beijing Qingke Biotechnology Co., Ltd. (Beijing, China) for synthesis.

Double-stranded sgRNAs with BbsI. endonuclease sites were synthesized by reacting at 95 °C for 10 min according to the following 10 μL annealing system (Table 3). The linearized PX458 plasmid was obtained by recovering the digested products after agarose gel electrophoresis in a water bath at 37 °C for 2 h (Table 4), and the linearized PX458 plasmid, after BbsI. endonuclease digestion, was ligated with double-stranded sgRNA overnight at 4 °C. The ligation product was then transformed into DH5α competent cells and, on the second day, monoclonal colonies were picked and placed in LB liquid to expand the culture. The bacterial solution was sent to Sanger to identify the vector sequence. After the three eukaryotic expression vectors were successfully constructed, they were named PX458-sgRNA109, PX458-sgRNA128, and PX458-sgRNA139, respectively. Then, the bacterial solution was expanded and cultured again. An endotoxic plasmid extraction kit was used to extract the correctly sequenced vector plasmid, and the concentration was determined and stored at −20 °C for subsequent cell transfection.

#### 2.5.3. sgRNA Cleavage Activity Assay

To confirm the cleavage activity of Cas9 protein in endogenous DNA sequences guided by sgRNAs designed at different sites, the target-specific sgRNA activity of PX458-sgRNA109, PX458-sgRNA128, and PX458-sgRNA139 vectors was evaluated by T7EI. enzyme digestion.

The specific sgRNA carried by the PX458 vector was introduced into third-generation fetal bovine fibroblasts (F3). The cells were recovered after 72 h of transfection and EGFP-positive single cells were selected via flow cytometry (SONY MA900, SONY, Tokyo, Japan) under FITC-A/SSC-A scattered light conditions. Then, the cells (cell amount greater than 2.0 × 10^6^) were collected, the genomic DNA was extracted, and the PCR gel recovery product was treated with T7EI enzyme and reacted at 37 °C for 30 min (Table 5). The reaction products were identified via electrophoresis of the enzyme digestion system by 2% agarose gel.

#### 2.5.4. Plasmid Amplification

The PX458-sgRNA139 vector with the highest sgRNA cleavage activity was selected and added to DH5α competent cells, gently aspirated and mixed, incubated on ice for 30 min, and placed on ice for 2 min immediately after a 42 °C water bath for 50 s. Next, we added an antibiotic-free LB culture medium, incubated the mixture at 37 °C for 1 h with shaking on a shaker, and then evenly applied it to an LB solid medium containing ampicillin and incubated it in a 37 °C incubator for 14 h. On the second day, single colonies were picked. The plasmid was first expanded in an LB liquid medium containing ampicillin and then extracted using an Omega Endotoxin-free Plasmid Extraction Kit and stored at −20 °C for later use.

### 2.6. Electroporation System Optimization and Edited Cell Transfection

To explore the growth state of the fetal bovine PRLR after electroporation at different editing sites, a fluorescent plasmid was used to optimize the electroporation system, and the cell state of fetal bovine sgRNA109 and sgRNA139 edited cells was observed after electroporation under optimized conditions.

Electroporation cups with 0.2 cm cuvette gaps were selected. When the fibroblast confluence reached 70–80% before transfection, the cells were trypsinized for 1 min, the cells were quickly pipetted with a pipette and aspirated, the supernatant was discarded by centrifugation at 800 r·min^−1^ for 10 min, and OPTI-MEM was used as the electroporation buffer and cell suspension (900 μL; the cell volume was about 1 × 10^6^ cells) and mixed well with the plasmid.

Fetal bovine sgRNA109 and sgRNA139 edited cells (F9) were transfected with the optimal electroporation system. After transfection, the cells were pipetted several times to mix well. Then, 300 μL of the transfected cells was seeded per well in a 6-well cell culture plate and cultured at 37 °C in a 5% CO_2_ cell culture incubator. The transfected cells were observed and photographed on the 5th day.

### 2.7. Screening of PRLR Gene-Edited Fetal Bovine Cells

#### 2.7.1. Flow Cytometry Sorting of Monoclonal Cells

After 72 h of recovery of growth performance, the electroporation-transfected cells seeded in 6-well plates were sequentially sorted via flow cytometry (SONY MA900) through FSC-A/SSC-A, FSC-A/FSC-H, and FITC-A/FSC-H channels, and the sorted cells were sorted into single-cell plates and expanded for culture.

#### 2.7.2. Gene Expression Analysis of Stable Transfection Cell Lines

After sorting the single-cell plates, the cells were cultured for 1–5 days until the cells grew to 2.5 × 10^6^. Then, the cell genomic DNA was extracted using a Blood/Cells/Tissue Genomic DNA Extraction Kit, and the extracted cell genomic DNA samples were identified by using specific primers (Table 6).

#### 2.7.3. Protein Expression Analysis of Stable Transfection Cell Lines

The cells were extracted from the total protein with a special extraction reagent for membrane proteins, without denaturation, mixed with 5× loading buffer, the proteins on each channel were separated by SDS-PAGE electrophoresis (10%), and the voltage was adjusted to 120 V for 1 h after confirming that the protein had run out of the stacking gel. Proteins were transferred to a polyvinylidene fluoride membrane for 30 min at 25 V and blocked with TBST containing 3% BSA for 4 h at room temperature. We diluted the primary antibody to the appropriate concentration according to the instructions and incubated the solution at 4 °C for 14 h. After incubation, the membrane was washed five times with TBST for 5 min each. Secondary antibodies were incubated for 1 h at room temperature. Then, we washed the membrane three times with TBST for 10 min each. After completion, the chemiluminescence solution was spread on the membrane and observed by glue.

### 2.8. Effect of PRLR Gene-Edited Cells on Embryonic Development of Fetal Cattle

#### 2.8.1. Somatic Cell Nuclear Transfer Experiments in Dairy Cows

Fetal bovine fibroblasts (F3) and gene-edited cells (F9) were selected as donor cells. A total of 122 dairy cows from the North Asia Youth Holstein Dairy Farm in Hohhot, Inner Mongolia Autonomous Region were selected. After slaughter, fresh bovine ovaries were collected. The oocytes with a full, rounded shape, uniform color, clear cytoplasm, and uniform diffusion of granule cells were extracted by the suction method using a 20 mL syringe, placed in a 4-well plate, cultured at 38.5 °C in a 5% CO_2_ incubator, and matured for 22 h in vitro.

Afterwards, the mature oocytes were treated with hyaluronidase for 5 min; the cumulus cells were removed and the mature oocytes of the second polar body were picked out. Then, the polar body of the mature oocyte and the surrounding cytoplasm were sucked out by the micromanipulator, the nucleus of the donor cells was injected into the enucleated oocytes, and the two cells were fused to form a reconstituted embryo by electrofusion. Then, the reconstructed embryo was activated by simulating the physiological signals after in vivo fertilization (the reconstructed embryo was activated in ionomycin for 5 min and then placed in 6DAMP). After 3 h, the reconstructed embryo was placed in a development medium to promote its development.

Finally, the activated, reconstituted embryos were placed in a cell culture incubator at 38.5 °C and 5% CO_2_ for seven days. Development indicators such as the cleavage rate and blastocyst rate were observed and recorded to evaluate the experimental effect.

#### 2.8.2. Statistics on Embryonic Development

After the reconstituted embryo was placed into the appropriate culture system, it was observed for seven consecutive days, with the development culture medium changed every other day. Development indicators such as the cleavage rate and blastocyst rate were observed and recorded, and the experimental effect was evaluated.

### 2.9. Statistical Analysis

Data analysis and graphical representation were performed using FlowJo (V10, BD Biosciences, Franklin Lakes, NJ, USA) and GraphPad Prism 9.0 software (Insightful Science, Boston, MA, USA). The statistical significance was set to *p* < 0.05.

Agarose gel electrophoresis was performed on the reaction products of T7EI, and the gray values of the electrophoresis images were calculated using ImageJ (1.8.0) to obtain the cleavage efficiencies of different sgRNAs. The formula for calculating the cutting efficiency is presented in Table 7.

## 3. Results

### 3.1. Tissue Culture and Identification

**Figure 2:**
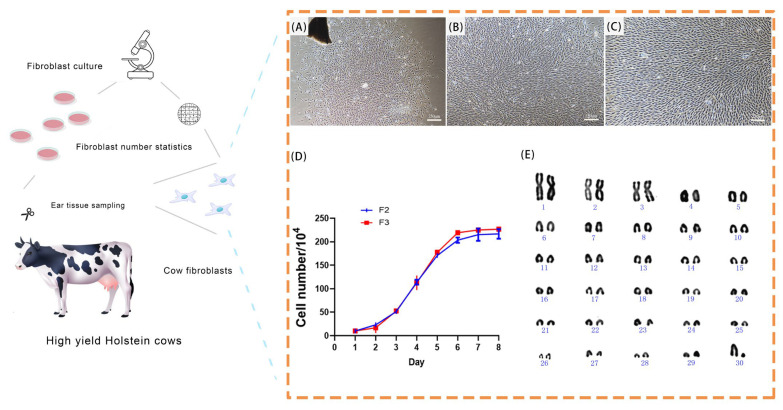
Culturing and identification of Holstein bovine fibroblasts. A depiction of the cell collection, culturing process, and statistical evaluation. (**A**) Primary fetal bovine fibroblast morphology (250 μm). (**B**) F2 fetal bovine fibroblast morphology (250 μm). (**C**) F3 fetal bovine fibroblast morphology (250 μm). (**D**) Growth curve showing the growth density of F2 and F3 cells. (**E**) Chromosome karyotype analysis of F3 cells.

In this study, ear tissue from one-month-old Holstein calves was utilized for the primary cell culture. By day 6 of culturing, spindle-shaped cells commenced their migration from the edges of the tissue blocks and expanded outward. By day 12, the cell confluence reached 80% (Figure 2A). During subsequent subcultures, the second-generation (F2) cells entered the logarithmic growth phase by day 3, achieving 80% confluence (Figure 2B). Similarly, the third-generation (F3) cells reached the logarithmic phase and 80% confluence by day 3 (Figure 2C). Both F2 and F3 exhibited typical growth trends indicative of healthy bovine fibroblasts (Figure 2D).

Post-subculturing, the cells maintained a stable spindle-shaped morphology with distinct contours and robust growth, confirming the successful establishment of fetal bovine fibroblast cultures.

### 3.2. Fetal Bovine sgRNA Design and PRLR Gene-Editing Activity Detection

**Figure 3:**
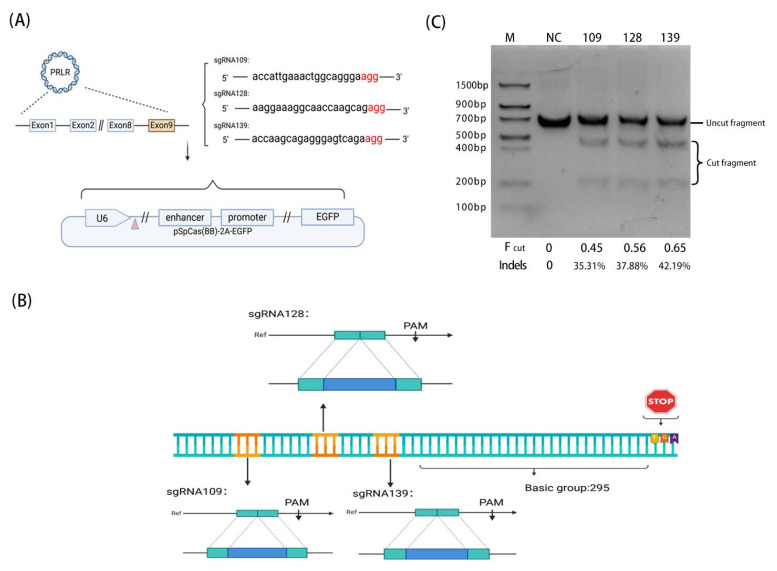
Construction of PRLR exon 9 sgRNA oligonucleotides into a targeting vector and the construction of a sgRNA plasmid sequence. (**A**) For PRLR exon 9, we designed three sequences, sgRNA109 sgRNA128, and sgRNA139, inserting the sgRNA in pSpCas (BB) after the U6 promoter plasmid construct; the PAM sequences are agg in red font (Gene name: PRLR. Gene location: Exon 9. Sequence direction: 5′ → 3′. Sequence size: 240 bp). (**B**) The relative positions of the three sgRNAs to the stop codon.The positions of the three sgRNAs (sgRNA109, sgRNA128, sgRNA139) designed by us are marked in the picture. There are 295 DNA base pair omitted between the three sgRNAs and the stop codon. (**C**) T7EI. Digestion electrophoresis results and ImageJ grayscale analysis results (M lane is Trans DNA Marker II. The NC lane was derived from a 690 bp size fragment of a fetal bovine fibroblast genome PCR amplification product transfected with a Cas9 expression vector without sgRNA insertion. Lane 109 is sgRNA 109, lane 128 is sgRNA 128, and lane 139 is sgRNA 139. Fcut represents the cleavage rate at which sgRNA-guided Cas9 cleaves genomic DNA, and Indels denotes the actual cleavage efficiency, which is also the proportion of heterozygous double-stranded DNA).

In this study, the breeding loci of genes related to the heat tolerance of Holstein cows were edited for the first time. Three sgRNAs with different loci were designed for the 50–240 bp fragment of exon 9 of the PRLR in dairy cows, and the editing activity was detected (Figure 3A).

In the experiment, pSpCas9(BB)-2A-EGFP (PX458, plasmid #48138) was used as the base vector. Through enzyme digestion, annealing, and ligation, the designed sgRNA109, sgRNA128, and sgRNA139 were inserted into the PX458 plasmid downstream of the U6 promoter, respectively. The results showed that the Fcuts of sgRNA109, sgRNA128, and sgRNA139 were 0.45, 0.56, and 0.65, respectively, and the Indels were 35.31%, 37.88%, and 42.19%, respectively. The activity of the three different sgRNAs guided Cas9 to cleave endogenous DNA in the following order, from largest to smallest, sgRNA139 > sgRNA128 > sgRNA109 (Figure 3B).

The results showed that the sgRNA-guided endogenous DNA cleavage of Cas9 protein could be realized at three different sites in fetal cattle transfected cells, and the sgRNA editing sites could be effectively designed. The actual cleavage efficiency of sgRNA139 was the highest, and that of sgRNA109 was the lowest.

In this experiment, a 240 bp segment from exon 9 of the PRLR was targeted for gene editing. Appropriate sgRNA sequences were designed to align with the gene-editing objectives. Employing the pSpCas9(BB)-2A-EGFP (PX458, plasmid #48138) as a foundational vector, a reconstruction vector specifically targeting exon 9 of the bovine PRLR was engineered through a series of enzyme digestion, annealing, and ligation procedures. The outcomes of the plasmid construction and enzyme digestion are depicted in the figure below.

### 3.3. Sites for Electroporation of Fetal Bovine PRLR Gene-Edited Cells

To explore the growth state of fetal bovine PRLR after electroporation at different editing sites, a fluorescent plasmid was used to optimize the electroporation system, and the cell state of fetal bovine sgRNA109 and sgRNA139 edited cells was observed after electroporation under optimized conditions.

**Figure 4:**
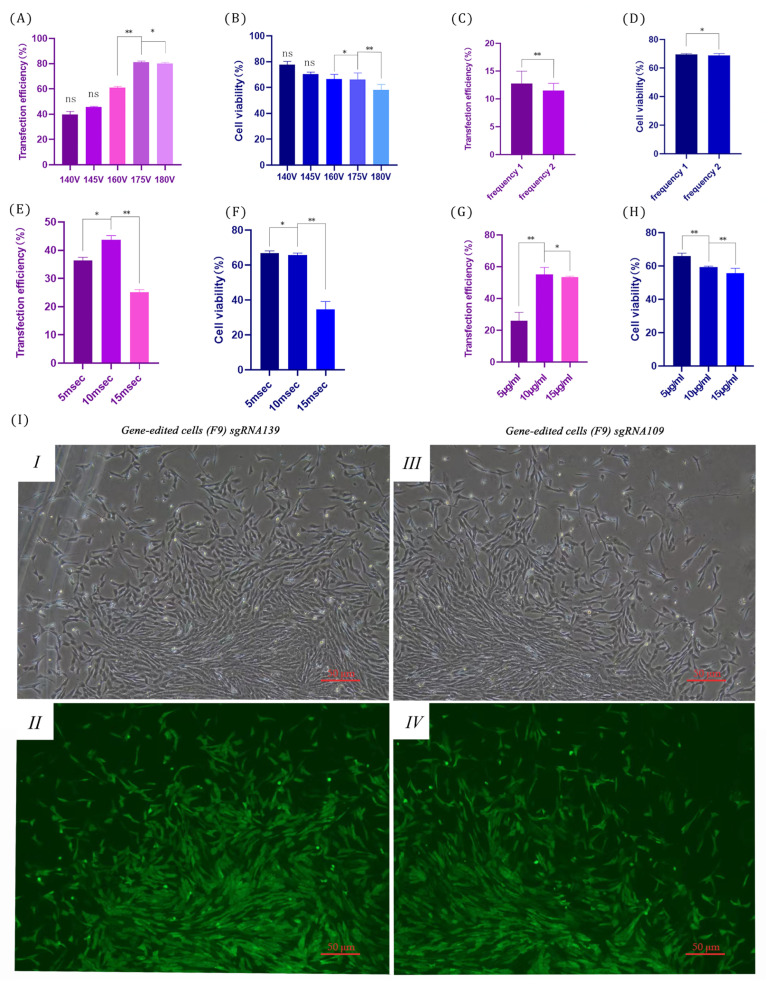
Plasmid transfection system optimization. (**A**) Transfection efficiency across different pulse voltages; (**B**) cell viability at various pulse voltages; (**C**) transfection efficiency with different pulse numbers; (**D**) cell viability with varying pulse numbers; (**E**) transfection efficiency under different pulse durations; (**F**) cell viability at various pulse durations; (**G**) transfection efficiency at different plasmid doses; (**H**) cell viability under various plasmid doses; (**I**) optimal electroporation system for editing cells ((**I**) sgDNA139 brightfield (50 μm) for gene-edited cells, (**II**) sgDNA139 EGFP field (50 μm) for gene-edited cells, (**III**) sgDNA109 brightfield (50 μm) for gene-edited cells, (**IV**) sgDNA109 EGFP field (50 μm) for gene-edited cells. The results are presented as the mean ± standard deviation (SD) of three independent experiments. One-way ANOVA with Bonferroni post hoc tests was used for statistical analysis (ns indicates *p* > 0.05, * *p* < 0.01, and ** *p* < 0.05).

The results showed that, during the optimization of the fluorometric electroporation system, the transfection efficiency was significantly different at 160 V and 175 V (*p* < 0.01), and between 175 V and 180 V (*p* < 0.05). There was a significant difference in cell activity between 160 V and 175 V (*p* < 0.05), and a significant difference between 175 V and 180 V (*p* < 0.01) (Figure 4A,B). The transfection efficiency was significantly different at 1 and 2 pulses (*p* < 0.01), as was the cell viability (*p* < 0.01) (Figure 4C,D). There were significant differences in staining efficiency and cell viability at 5 msec and 10 msec (*p* < 0.05), and extremely significant differences between 10 msec and 15 msecs (*p* < 0.01) (Figure 4E,F). The transfection efficiency was significantly different between 5 μg/mL and 10 μg/mL (*p* < 0.01), and between 10 μg/mL and 15 μg/mL (*p* < 0.05). The difference in cell activity was extremely significant at 5 μg/mL and 10 μg/mL plasmid DNA doses (*p* < 0.01), and between 10 μg/mL and 15 μg/mL (*p* < 0.01) (Figure 4F,G).

In summary, when there was one pulse with a voltage of 175 V and a duration of 5 msec, the plasmid DNA dose was 10 μg/mL, and the cell transfection efficiency and cell viability were the best. The transfection efficiency of sgRNA139 and sgRNA109 in gene-edited cells (F9) reached more than 90% under the sub-transfection system, so it could be used for follow-up research (Figure 4I).

### 3.4. Screening and Identification of Fetal Bovine PRLR Gene-Edited Cells

**Figure 5:**
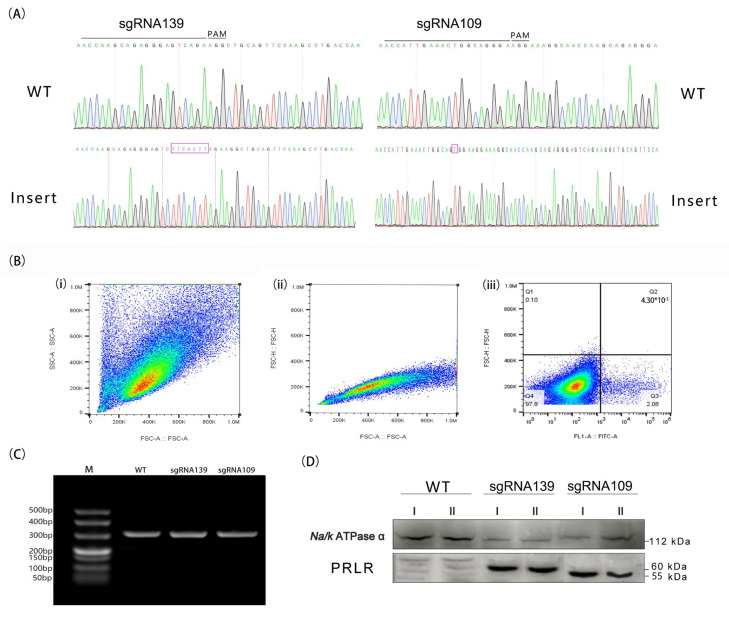
Screening of gene-edited cells for stable transfection. (**A**) Sanger sequencing map of the genomic DNA of the edited cells. The editing window is located at the position of the first three bases in front of the PAM sequence, sgRNA139 insert “TTCACTT”; sgRNA109 insert “T”. (**B**) Flow cytometry sorting workflow: (**i**) the main cell population was identified through the FSC-A/SSC-A channel; (**ii**) adhesion aggregates were excluded, and single cells were isolated through the FSC-A/FSC-H channel; and (**iii**) positive cells were sorted through the FITC-A/FSC-H channel, with 2.08% positive cells identified (Q3). (**C**) Agarose gel (1%) electrophoresis results for PCR-amplified single cells. (**D**) Western blot for protein expression detection (internal control: Na/K ATPα (112 kDa), sgRNA139 (60 kDa), sgRNA109 (55 kDa)).

In the study, flow cytometry was used to sort GFP-positive cells from the electroporated cells. After the electroporated cells recovered their growth performance for 72 h, the main cell population was screened through the FSC-A/SSC-A channel (Figure 5(Bi)) using a flow cytometer. Then, the cells were sorted to remove cell aggregates through the FSC-A/FSC-H channel (Figure 5(Bii)). Subsequently, 2.08% of GFP-positive cells were obtained through the FITC-A/FSC-H channel (Figure 5(Biii)). After that, the GFP-positive cells were sorted into single cells in 96-well plates and expanded in culture.

A PCR results showed that the PCR products at sgRNA109 and sgRNA139 appeared in the 330 bp lane. (Figure 5B), and the single-cell expansion was completed after screening. A protein analysis showed that the protein at sgRNA109 was expressed around 55 kDa, while the protein at sgRNA 139 was expressed at 60 kDa. The sgRNA 109 locus induces the Cas9 protein to cleave fetal bovine endogenous DNA, stopping codon expression in advance of self-repair.

The results showed that the sgRNA109 and sgRNA139 loci guided the Cas9 protein to cleave the endogenous DNA of fetal cattle with different cleavage effects. The expression sites of stop codons in the self-repair process of the stable cell line were confirmed to be inconsistent through gene- and protein-level analyses, and all of them were smaller than those in the control group (*p* < 0.05).

### 3.5. Embryo Development Statistics

This study evaluated the impact of using sgRNA109 and sgRNA139 sites to guide the Cas9 protein to cut the endogenous DNA of fetal bovine to generate stable cell lines for embryo development through somatic cell nuclear transfer experiments.

**Figure 6:**
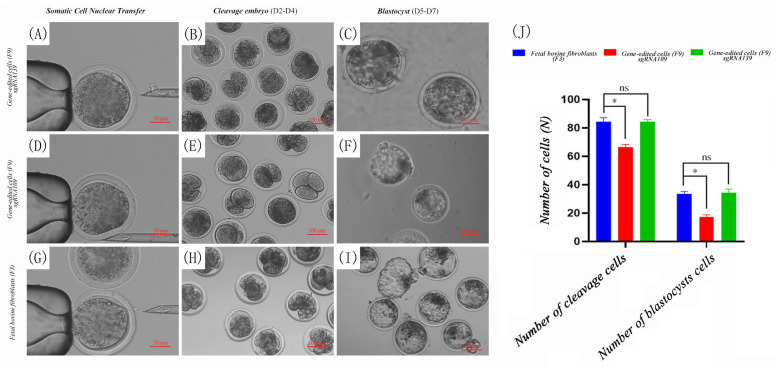
Somatic cell nuclear transfer experiment and embryonic development (**A**) Gene-edited cell sgDNA139 (F9) nuclear transfer enucleation process (50 μm). (**B**) Gene-edited cell sgDNA139 (F9) cleavage stage embryo (development time: D2–D4, 100 μm). (**C**) Gene-edited cell sgDNA139 (F9) blastocyst (development time: D5–D7, 50 μm). (**D**) Gene-edited cell sgDNA109 (F9) nuclear transfer enucleation process (50 μm). (**E**) Gene-edited cell sgDNA109 (F9) cleavage stage embryo (development time: D2–D4, 100 μm). (**F**) Gene-edited cell sgDNA109 (F9) blastocyst (development time: D5–D7, 100 μm). (**G**) Fibroblast (F3) somatic cell nuclear transfer enucleation process (50 μm). (**H**) Fibroblast (F3) cleavage stage embryo (development time: D2–D4, 100 μm). (**I**) Fibroblast (F3) blastocyst (development time: D5–D7, 100 μm). (**J**) Statistics of cleavage rate and blastocyst rate of gene-edited cells (number: N) (* Indicates *p* < 0.05, ns indicates no significant difference).

After the oocytes matured for 22 h, somatic cell nuclear transfer experiments were conducted on stable cell lines with different editing sites. After the experiments were completed, the reconstructed embryos were cultured for development. The cells of each group reached the cleavage stage of the reconstructed embryo at 2–4 days (Figure 6B,E,H), with uniform cleavage of blastomeres, smooth zona pellucida, and good development; they reached the blastocyst stage of the reconstructed embryo at 5–7 days (Figure 6C,F,I), with tight and uniform cell arrangement, moderate blastocoel size, clear boundaries, and a regular appearance that could be visually perceived, ready for hatching at any time.

Through continuous seven-day observation and recording, the results showed that, after 300 fibroblasts (100 each time) (F3) were subjected to somatic cell nuclear transfer experiments three times, the cleavage rate was 81 ± 0.2% and the blastocyst rate was 36 ± 0.3%. After 300 stable cells with the sgRNA109 site (F9) were subjected to somatic cell nuclear transfer experiments three times, the cleavage rate was 70 ± 0.2% and the blastocyst rate was 26 ± 0.3%. After 300 stable cells with the sgRNA139 site (F9) were subjected to somatic cell nuclear transfer experiments three times, the cleavage rate was 81 ± 0.2% and the blastocyst rate was 35 ± 0.1%.

The use of edited cells at different sites as donor cells for somatic cell nuclear transfer had differential effects on the later development of embryos. The sgRNA109 site was significantly lower than normal cells in terms of both the cleavage rate and blastocyst rate indicators (*p* < 0.01), while the sgRNA139 site showed no significant difference in cleavage rate (*p* > 0.05) and had a lower blastocyst rate than normal fibroblasts; however, the difference was not significant (*p* > 0.05). This effectively indicates that, for donor cells, efficient and feasible sgRNA design is necessary, which will directly affect the later development of embryos (Figure 6J).

## 4. Discussion

CRISPR/Cas9, widely used by researchers, comprises the Cas9 enzyme and a sgRNA, which collectively mediate the cleavage of target site sequences, inducing DNA double-strand breaks and thus activating the pathways for endogenous repair. This process introduces random mutations during repair [13]. Undoubtedly, enhancing editing efficiency is the most critical factor in the editing system, as highly efficient editing provides a powerful tool for research in gene function analysis and disease treatment [14]. At present, one of the most effective methods to improve editing efficiency is designing sgRNAs that are highly site-specific. The outcomes of gene editing can be better controlled by sgRNA, which enables the precise modification of specific genes.

In breeding dairy cattle that are resistant to heat stress, studies have confirmed that variations in sgRNA design influence the repair of endogenous DNA during gene editing, which causes stop codons to occur with different timing [15]. Moreover, single mutations in bovine PRLR have been shown to have significant genetic impacts on the length of hair and its structural characteristics [16]. In particular, the slick phenotype, characterized by a smooth and short-haired coat, has been shown to result from a frameshift mutation in the last exon of PRLR, where a portion of its cytoplasmic domain is truncated [17]. To further investigate the impact of PRLR-edited cells on embryonic development, this study designs various sgRNAs targeting the 50–204 bp region of the ninth exon, guiding the Cas9 protein to induce the cleavage of endogenous DNA for self-repair. Subsequently, it screens for stably transfected cell lines and investigates the effects of these cell lines on embryonic development through SCNT experiments.

This study is the first to design diverse sgRNAs targeting the 50–204 bp region of the ninth exon in bovine PRLR. Using pSpCas9(BB)-2A-EGFP (PX458, plasmid #48138) as the base vector, we employed T7 endonuclease I (T7EI) to assess the cleavage activity of different sgRNAs and analyzed PCR products amplified from the target gene-edited sites. The results revealed that the Fcut values for sgRNA109, sgRNA128, and sgRNA139 were 0.45, 0.56, and 0.65, respectively, and their corresponding Indels rates were 35.31%, 37.88%, and 42.19%, respectively. The activity of the three sgRNAs guiding Cas9 to cleave endogenous DNA was ranked as follows: sgRNA139 > sgRNA128 > sgRNA109. These findings demonstrate that, during electroporation, all sgRNAs successfully guided the Cas9 protein to cleave endogenous DNA. Furthermore, sgRNA139 exhibited the highest cleavage efficiency, while sgRNA109 demonstrated the lowest cleavage efficiency.

We conducted electroporation with sgRNA109 and sgRNA139 and achieved a high transfection efficiency (>90%), as observed through cell morphology. Subsequent identification and analysis of the edited cells revealed that sgRNA109 and sgRNA139 guided the Cas9 protein to cleave endogenous DNA and produced diverse cleavage effects. Analysis at both the gene and protein levels confirmed that stable transfection cell lines exhibited different timing of stop codons during self-repair. In this study, the repair of endogenous DNA guided by sgRNAs resulted in PRLR of different sizes: sgRNA139 formed a 60 kDa protein, while sgRNA109 formed a 55 kDa protein. Western blotting demonstrated significant differences in gray values between edited cells and the control group (*p* < 0.05). We hypothesize that the proteins underwent various structural changes during repair in edited cells. Firstly, proteins with strong hydrophilicity dissolve more readily in water during sample preparation. This allows us to obtain a greater quantity of proteins in their native state. Such proteins can bind more effectively to antibodies, intensifying the color of the bands. We hypothesize that during heat stress, dairy cows experience changes in the state of certain proteins. Responding to the adverse environment, these proteins interact with each other at the molecular level to adapt [18]. Moreover, proteins may have undergone post-translational glycosylation. After protein synthesis, sugar molecules are covalently attached to specific amino acid residues of the protein. Glycosylated proteins significantly increase in molecular weight, thereby regulating the metabolic functions of dairy cattle and affecting cellular performance in physiological processes such as heat tolerance. This is closely related to the mechanisms of cellular response to heat stress. As mentioned earlier in research on gene editing for heat-tolerant dairy cattle, these influence the physiological functions and related traits of dairy cattle [19]. This allows for the expression of protein variants that are more adapted to high-temperature environments, optimizing physiological functions under heat stress, maintaining normal metabolic rates at high temperatures, and ensuring the supply of materials and energy needed for embryonic development [20].

To further validate the impact of PRLR-edited cells on embryonic development, we conducted SCNT experiments using stably transfected cell lines. The results of embryonic development indicated that donor cells from both the experimental and control groups could produce embryos with full and abundant inner cell masses, demonstrating strong differentiation potential and providing a solid foundation for developing organ systems in the fetal stage. However, using different sgRNAs as donor cells led to differential effects on late embryonic development [21]. The cleavage and blastocyst rates of sgRNA109 were significantly lower than in normal cells (*p* < 0.01), while sgRNA139 exhibited no significant difference in cleavage rate (*p* > 0.05). In addition, the blastocyst rate was lower than in normal fibroblasts, but not significantly different (*p* > 0.05). We hypothesize that the differential amino acid sequences resulting from protein translation at sgRNA139 and sgRNA109 play a crucial regulatory role in fetal development, thereby influencing embryonic cleavage and blastocyst development.

The study further confirms that designing efficient and feasible sgRNAs for donor cells is crucial, as this directly impacts late embryonic development. The structure of PRLR comprises an extracellular domain, a transmembrane domain, and an intracellular domain. Gene editing can induce conformational changes in the receptor, thereby activating intracellular signaling pathways, promoting alterations in physiological processes such as cell proliferation, differentiation, metabolism, and secretion, and regulating physiological responses to heat stress. Further investigation into the effects of PRLR-edited cells on bovine embryonic development is of significant importance. Future studies will focus on selecting appropriate targets for gene editing, optimizing editing techniques and establishing effective systems for screening and evaluating cells. Through the application of these methods, bovine embryonic development can be enhanced, thereby providing valuable guidance for the application of gene-editing techniques and the breeding of heat-stress-resistant dairy cattle in China.

## 5. Conclusions

In this study, three sgRNAs (sgRNA139, sgRNA128, and sgRNA109) targeting the PRLR in fetal bovines were successfully designed using the CRISPR/Cas9 system, achieving efficient gene editing. The results showed that sgRNA139 exhibited the highest DNA repair efficiency (Fcut = 0.65, Indels = 42.19%), while sgRNA109 had the lowest repair efficiency (Fcut = 0.45, Indels = 35.31%), indicating that the sequence specificity of sgRNAs and their binding affinity to the target DNA may influence editing efficiency. Through somatic cell nuclear transfer (SCNT) experiments, the impact of different editing sites on embryonic development was evaluated. The results revealed that sgRNA109 significantly reduced the cleavage rate and blastocyst rate (*p* < 0.01), while sgRNA139 had no significant effect on the cleavage rate (*p* > 0.05), although its blastocyst rate was slightly lower than that of the control group (*p* > 0.05). The PRLR plays a critical role in embryonic development, as its encoded prolactin receptor regulates physiological processes such as cell proliferation, differentiation, and metabolism, influencing the heat tolerance of dairy cattle. This study provides important theoretical and practical guidance for the molecular breeding of heat-tolerant traits in dairy cattle. Future research could further optimize gene editing techniques and explore additional gene targets related to heat tolerance, offering scientific insights for breeding dairy cattle adapted to high-temperature environments.

## Figures and Tables

**Table 1:** sgRNA sequence.

sgRNA of Number	sgRNA Sequence (PAM:NGG)
109	ACCATTGAAACTGGCAGGGA-AGG
128	AAGGAAAGGCAACCAAGCAG-AGG
139	ACCAAGCAGAGGGAGTCAGA-AGG

**Table 2:** The oligonucleotide sequence corresponding to the target sequence.

Number	Top Guide Oligo (5′-3′)	Bottom Guide Oligo (5′-3′)
109	CACCACCATTGAAACTGGCAGGGA	AAACTCCCTGCCAGTTTCAATGGT
128	CACCAAGGAAAGGCAACCAAGCAG	AAACCTGCTTGGTTGCCTTTCCTT
139	CACCACCAAGCAGAGGGAGTCAGA	AAACTCTGACTCCCTCTGCTTGGT

**Table 3:** sgRNA oligonucleotide annealing system.

sgRNA	Volume (μL)
sgRNA-Top Oligo	5
sgRNA-Bottom Oligo	5

**Table 4:** PX458 Linearized reaction system.

Component	Volume (μL)
pSpCas9(BB)-2A-GFP	2 μg
Bbs I-HF	1
10 × rCutSmart Buffer	5
ddH_2_O	to 50

**Table 5:** Primer for T7EI-PRLR-amplicon.

Primer Name	Primer Sequence (5′-3′)	Amplicon Length
T7EⅠ-PRLR-amplicon	F:GACTGCGAGGACTTGCTGAT	790 bp
	R:ACAGAGTCAGGTTTTGCGCT	

**Table 6:** Primer list for real-time quantitative polymerase chain reaction (PCR).

Ampliconname	Forward Primer	Reverse Primer	Length (bp)
PRLR	CCACAACATTGCTGACGTGT	TACTCCTTGCTGGCTTCAGG	400

**Table 7:** Formula for calculating cutting efficiency.

Name	Formula
Cutting efficiency	Fcut = sum of gray values of two strips formed by enzyme digestion/sum of gray values of three strips in this lane
The actual cutting efficiency of Cas9 protein at the target site	Indel% = 1 − (1 − Fcut) 1/2 × 100

## Data Availability

Data are contained within the article.
